# CAPS-1 requires its C2, PH, MHD1 and DCV domains for dense core vesicle exocytosis in mammalian CNS neurons

**DOI:** 10.1038/s41598-017-10936-4

**Published:** 2017-09-07

**Authors:** Linda van Keimpema, Robbelien Kooistra, Ruud F. Toonen, Matthijs Verhage

**Affiliations:** 1 Department of Functional Genomics, Center for Neurogenomics and Cognitive Research, VU University, 1081 HV Amsterdam, The Netherlands; 2 Sylics (Synaptologics BV), PO box 71033, 1008 BA Amsterdam, The Netherlands

## Abstract

CAPS (calcium-dependent activator protein for secretion) are multi-domain proteins involved in regulated exocytosis of synaptic vesicles (SVs) and dense core vesicles (DCVs). Here, we assessed the contribution of different CAPS-1 domains to its subcellular localization and DCV exocytosis by expressing CAPS-1 mutations in four functional domains in CAPS-1/-2 null mutant (CAPS DKO) mouse hippocampal neurons, which are severely impaired in DCV exocytosis. CAPS DKO neurons showed normal development and no defects in DCV biogenesis and their subcellular distribution. Truncation of the CAPS-1 C-terminus (CAPS Δ654-1355) impaired CAPS-1 synaptic enrichment. Mutations in the C2 (K428E or G476E) or pleckstrin homology (PH; R558D/K560E/K561E) domain did not. However, all mutants rescued DCV exocytosis in CAPS DKO neurons to only 20% of wild type CAPS-1 exocytosis capacity. To assess the relative importance of CAPS for both secretory pathways, we compared effect sizes of CAPS-1/-2 deficiency on SV and DCV exocytosis. Using the same (intense) stimulation, DCV exocytosis was impaired relatively strong (96% inhibition) compared to SV exocytosis (39%). Together, these data show that the CAPS-1 C-terminus regulates synaptic enrichment of CAPS-1. All CAPS-1 functional domains are required, and the C2 and PH domain together are not sufficient, for DCV exocytosis in mammalian CNS neurons.

## Introduction

Calcium-dependent activator protein for secretion (CAPS) is an important regulator of dense core vesicle (DCV) exocytosis. In *C. elegans* null mutants of the CAPS homolog UNC-31, DCV exocytosis is impaired and DCV docking reduced^1–3^. Also in *Drosophila* CAPS (dCAPS) null mutants, DCV exocytosis in neuromuscular junctions is impaired and DCVs accumulate in synaptic terminals^4^. Mammals express two CAPS isoforms, CAPS-1 and CAPS-2, mainly in neuronal and endocrine cells^5–7^. Deletion of CAPS-1 and CAPS-2 expression (CAPS DKO) severely reduces DCV exocytosis in mammalian neurons and exocytosis of secretory granules in chromaffin cells^8–11^. This reduced DCV exocytosis is rescued by re-expression of CAPS-1 or CAPS-2 in chromaffin cells^9, 10, 12^ or CAPS-1 in neurons^8^. This suggests a redundant function of the two isoforms in vesicle exocytosis, although shRNA mediated knockdown of CAPS-1 reduces DCV exocytosis in hippocampal neurons^13^. In addition, CAPS-1 and CAPS-2 are also important for synaptic vesicle exocytosis. In CAPS DKO neurons, evoked SV exocytosis, readily releasable pool size and the number of docked SVs are severely impaired^14, 15^. *Drosophila* dCAPS null mutants show impaired SV release in the neuromuscular junction and accumulation of vesicles in synaptic terminals^4^. Hence, CAPS proteins are important regulators of both DCV and SV exocytosis.

CAPS proteins contain multiple functional domains. The C2 domain^16^ is involved in CAPS dimerization^17^. The pleckstrin homology (PH) domain of CAPS-1 binds to phospholipids and is essential for CAPS interaction with the plasma membrane^4, 16, 18^. CAPS-1 binds to SNARE proteins via Munc13 homology domain-1 (MHD1)^19–21^. Finally, the C-terminal part of CAPS-1 appears to be important for CAPS-1 interaction with DCVs in PC12 cells^16^. Mutations in these domains interfere with CAPS-1 function in calcium-dependent DCV exocytosis in PC12 cells^6, 16, 21–24^ and DCV exocytosis in *C. elegans* ^25^. However, a natural CAPS-2 splice isoform, which lacks the MHD1 and DCV domains, rescues exocytosis in CAPS DKO chromaffin cells^10^. Hence, there is no consensus on the importance of the CAPS protein domains for DCV exocytosis and current knowledge of CAPS protein domain function in mammalian CNS neurons is absent.

In this study, we analyzed the function of CAPS-1 domains by expressing domain mutants on a CAPS DKO null mutant background. Truncation of the CAPS-1 C-terminus, harboring the MHD1 and DCV domains, impaired CAPS-1 enrichment at synapses, but mutations in the C2 or PH domain did not. CAPS DKO neurons showed a drastic decrease in DCV release probability, which was rescued by expression of wild type CAPS-1 but not CAPS-1 C2 or PH domain mutants or the C-terminal truncation. In addition, deletion of both CAPS proteins affected DCV exocytosis more than SV exocytosis. Together, our study shows that all CAPS-1 functional domains are essential for DCV exocytosis in mammalian CNS neurons.

## Results

### CAPS-1 C-terminal truncation, but not C2 and PH domain mutations, influence CAPS-1 enrichment at synapses

CAPS-2 has been implicated in neuronal development. Over-expressed CAPS-2 promotes cell survival and deletion of CAPS-2 expression impairs dendritic arborization of mouse cerebellar Purkinje cells^26, 27^. To test if CAPS DKO neurons show developmental defects, which could influence the outcome of our functional assays, we analyzed neuronal development in hippocampal CAPS DKO neurons compared to CAPS-2 KO control neurons. During *in vitro* development (days *in vitro* (DIV) 2–14), total neurite length and number of synaptotagmin 1 (syt 1) positive puncta increased (neurite length DIV 2: 0.38 ± 0.03 mm; DIV 14: 3.44 ± 0.41 mm; syt1 positive puncta DIV 2: 22 ± 4.6; DIV 14: 809 ± 112, all data and statistics in Table S1; Figure S1a,b). No difference was observed between CAPS DKO and control neurons at any of the time points (neurite length CAPS DKO DIV 2: 0.41 ± 0.06 mm; DIV 14: 3.38 ± 0.37 mm; syt1 positive puncta CAPS DKO DIV 2: 27 ± 6.5; DIV 14: 862 ± 117, all data and statistics in Table S1; Figure S1c). Hence, CAPS DKO neurons show no additional developmental defects *in vitro* compared to the ones reported for CAPS-2 KO neurons.

**Figure 1:**
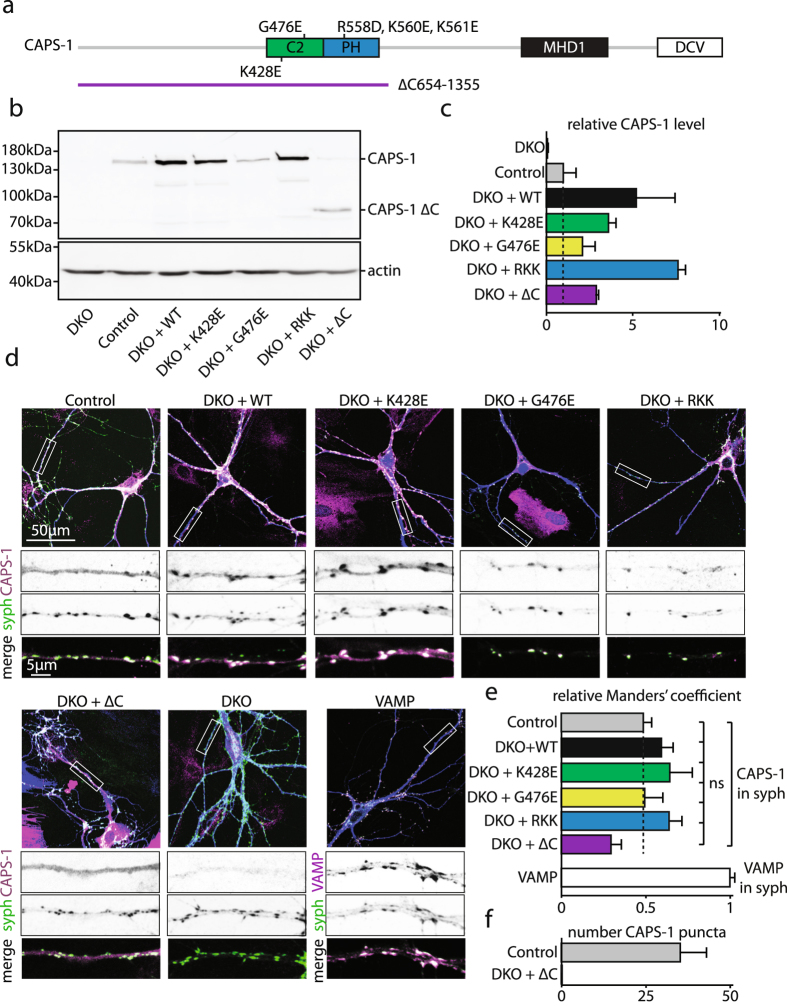
C-terminal truncation decreases CAPS-1 synaptic enrichment. (**a**) Schematic representation of mouse CAPS-1, showing the C2 domain (397–516)^16, 17^, PH domain (516–632)^16, 17^, MHD domain (933–1113)^19, 20^, and DCV domain (1220–1355)^16^. Numbers represent amino acid residues. Indicated are the CAPS-1 C2 domain mutants (K428E and G476E), the CAPS-1 PH domain mutant (R558D/K560E/K561E, RKK) and C-terminal truncation (Δ654–1355, ΔC). (**b**) Western blot of CAPS DKO cortical neurons infected with wild type or mutant CAPS-1 constructs and CAPS-2 KO control neurons (control). Actin was used as loading control, gel was cropped (full-length gel presented in Figure S2). (**c**) Quantification of relative CAPS-1 level in control neurons of two independent western blots of CAPS DKO cortical neurons infected with wild type or mutant CAPS-1 constructs and control neurons. CAPS-1 level was corrected for protein loading (using actin levels). (**d**) Representative images of CAPS DKO hippocampal neurons infected with WT, K428E, G476E, RKK, ΔC and control neurons, stained with dendrite marker (MAP2, blue), CAPS-1 (magenta) and synaptophysin 1 (syph, green). Boxed areas are enlarged at the bottom. (**e**) Manders’ coefficient of CAPS-1 in synaptophysin (syph), relative to colocalization of VAMP in syph, in CAPS DKO hippocampal neurons infected with wild type or mutant CAPS-1 constructs and control neurons. One-way ANOVA (CAPS conditions): p = 0.067 (not significant, ns). (**f**) Number of CAPS-1 puncta in control and DKO + ΔC neurons. Detailed information (average, SEM, n and detailed statistics) is shown in Table S1.

To test the function of CAPS-1 C2, PH and C-terminal domains in DCV exocytosis in CNS neurons, we expressed CAPS-1 mutants of the C2 domain (K428E and G476E^22^), the PH domain (R558D/K560E/K561E; RKK)^16^ and a CAPS-1 mutant with a truncated C-terminus (Δ654–1355; ΔC) in CAPS DKO neurons (Fig. 1a). Neurons from CAPS-2 KO littermates were used as controls as in previous studies, since deletion of CAPS-2 does not significantly alter SV or DCV exocytosis in the hippocampal neurons used in this study^8, 14^. All CAPS-1 mutants were expressed at least as high as endogenous CAPS-1 (control = 1.00 ± 0.74, CAPS-1 mutants ≥ 2.08 ± 0.79, CAPS DKO: 0.05 ± 0.04, all data in Table S1, Fig. 1b,c). Neurons were stained for dendritic marker MAP2, the synaptic marker synaptophysin 1 and CAPS-1 at DIV 10–14 (Fig. 1d). Endogenous CAPS-1 is present in the cytosol and neuritic puncta, which often co-localize with a presynaptic marker^8^. To test if CAPS-1 mutations affected CAPS-1 localization we analyzed their co-localization with the synapse marker synaptophysin 1. The C2 and PH domain mutants showed a similar synaptic accumulation as wild type CAPS-1 (relative Manders’ coefficient of control: 0.48 ± 0.05, DKO + WT: 0.61 ± 0.05, DKO + K428E: 0.66 ± 0.07, DKO + G476E: 0.48 ± 0.12, DKO + RKK: 0.64 ± 0.08), but the C-terminal truncation mutant showed a decreased synaptic localization (relative Manders’ coefficient of DKO + ΔC: 0.31 ± 0.05, all data and statistics in Table S1, Fig. 1e) and no CAPS-1 puncta (number CAPS-1 puncta control: 34.8 ± 7.7, DKO + ΔC: 0.0 ± 0.0, Fig. 1f). Hence, CAPS-1 C2 and PH domain mutations and C-terminal truncation do not prevent stable expression in neurons. The C2 and PH domain mutations do not alter subcellular localization, but truncation of the C-terminus diminishes synaptic enrichment.

### CAPS-1 C2, PH, MHD1 and DCV domains are required for CAPS-1 function in neuronal DCV exocytosis

**Figure 2:**
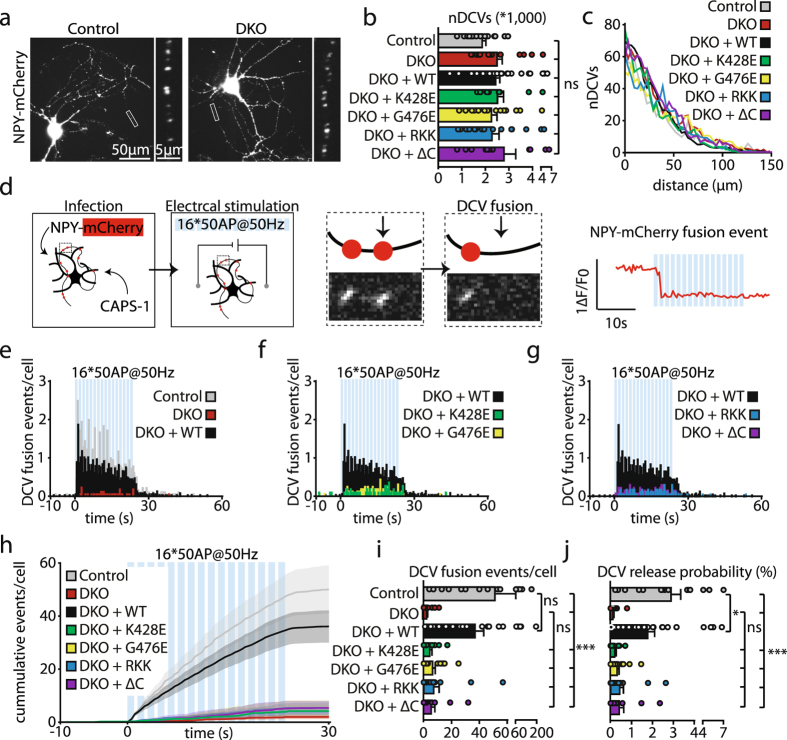
CAPS-1 C2 and PH domain and C-terminal truncation mutants do not support neuronal DCV exocytosis. (**a**) Representative images of neuronal DCV labeling with NPY-mCherry in CAPS-2 KO (control) and CAPS DKO neurons. Boxed area is enlarged on the right. (**b**) Number of DCVs in CAPS DKO hippocampal neurons infected with wild type or mutant CAPS-1 constructs and control neurons. One-way ANOVA: p = 0.42 (not significant, ns). (**c**) Sholl analysis of the number of DCVs in distal neurites of CAPS DKO neurons infected with wild type or mutant CAPS-1 constructs and control neurons. (**d**) Schematic representation of the method to measure neuronal DCV exocytosis. Neurons, co-infected with NPY-mCherry and CAPS-1, are stimulated with 16 trains of 50 AP at 50 Hz (blue bars), which induces DCV exocytosis (sudden disappearance of a fluorescent punctum, middle panels). Representative trace of DCV exocytosis is depicted on the right. (**e**–**g**) Average DCV exocytosis events per cell before, during and after stimulation in (**e**) control, CAPS DKO and DKO + WT, (f) DKO + WT, DKO + K428E and DKO + G476E and (**g**) DKO + WT, DKO + RKK and DKO + ΔC neurons. (**h**) Cumulative plot of DCV exocytosis events in CAPS DKO neurons infected with wild type or mutant CAPS-1 constructs and control neurons. Shaded area represents SEM. (**i**) Average DCV exocytosis events per cell in CAPS DKO neurons infected with wild type or mutant CAPS-1 constructs and control neurons. One-way ANOVA: p = 5.9 *10^−9^ (***); post-hoc Dunnett’s test: control vs DKO + WT: p = 0.27 (ns), control vs DKO (+CAPS-1 mutants): p ≤ 8.5 *10^−5^ (***), DKO vs DKO + CAPS-1 mutants: p ≥ 0.96 (ns). (**j**) DCV release probability in CAPS DKO neurons infected with wild type or mutant CAPS-1 constructs and control neurons. One-way ANOVA: p = 2.2 *10^−8^ (***); post-hoc Dunnett’s test: control vs DKO + WT: p = 0.045 (*), control vs DKO (+CAPS-1 mutants): p ≤ 1.9 *10^−4^ (***), DKO vs DKO + CAPS-1 mutants: p ≥ 0.98 (ns). Detailed information (average, SEM, n and statistics) is shown in Table S1.

Expression of full-length wild type CAPS-1 restores DCV exocytosis in CAPS DKO neurons^8^. Here, we assessed whether CAPS-1 C2 (K428E and G476E) or PH (RKK) domain and C-terminal truncation (ΔC) mutants support DCV exocytosis in CAPS DKO neurons. To visualize DCV exocytosis we used an established DCV-reporter, neuropeptide Y (NPY)-mCherry^8, 28, 29^ (Fig. 2a). CAPS DKO neurons were co-infected with NPY-mCherry (5–6 days before imaging) and wild type or mutant CAPS-1 at DIV 0–1. The total number of DCV labeled with this reporter (control: 1.9 ± 0.1 *10^3^, DKO: 2.3 ± 0.2 *10^3^, DKO + WT: 2.4 ± 0.2 *10^3^, DKO + K428E: 2.5 ± 0.3 *10^3^, DKO + G476E: 2.3 ± 0.3 *10^3^, DKO + RKK: 2.3 ± 0.3 *10^3^, DKO + ΔC: 2.8 ± 0.5 *10^3^, all data and statistics in Table S1, Fig. 2b) and their location along the neurites was similar in all groups (Fig. 2c). We applied electrical stimulation, 16 trains of 50 action potentials (AP) at 50 Hz, to trigger DCV exocytosis^8, 28, 29^. DCV exocytosis, characterized by a sudden disappearance of fluorescent NPY-mCherry puncta in dendrites and axons, was measured at DIV 9–15 (Fig. 2d). In CAPS DKO neurons infected with any of the mutants and in control neurons, exocytosis occurred primarily during electrical stimulation, with only a small fraction of the events before or after stimulation (Fig. 2e–h). The average number of DCV exocytosis events in CAPS DKO neurons (2.1 ± 0.8) was 96% lower compared to control neurons (51 ± 9.7), as observed before^8^. Expression of wild type CAPS-1 restored the number of DCV exocytosis events (36.9 ± 6.3). Neither of the mutants could increase the number of exocytosis events (DKO + K428E: 4.3 ± 1.7, DKO + G476E: 6.4 ± 1.9, DKO + RKK: 8.8 ± 3.8, DKO + ΔC: 5.4 ± 2.7, all data and statistics in Table S1, Fig. 2i), and showed similar exocytosis kinetics as DKO neurons (Fig. 2e–h). The DCV release probability, defined as the number of exocytosis events/total number DCVs per cell, was 2.9 ± 0.55% in control neurons and 1.8 ± 0.33% in CAPS DKO neurons rescued with wild type CAPS-1, but less than 0.5% in CAPS-1 C2 (DKO + K428E: 0.20 ± 0.07%, DKO + G476E: 0.31 ± 0.10%), PH domain (DKO + RKK: 0.43 ± 0.18%) or C-terminal truncation (DKO + ΔC: 0.41 ± 0.22%) mutants (all data and statistics in Table S1, Fig. 2j). These data show that the integrity of the CAPS-1 C2 and PH domains and presence of the CAPS-1 C-terminus are all required for the function of CAPS-1 in DCV exocytosis in mammalian CNS neurons.

### CAPS-1 deletion affects DCV exocytosis more than SV exocytosis upon intense stimulation

**Figure 3:**
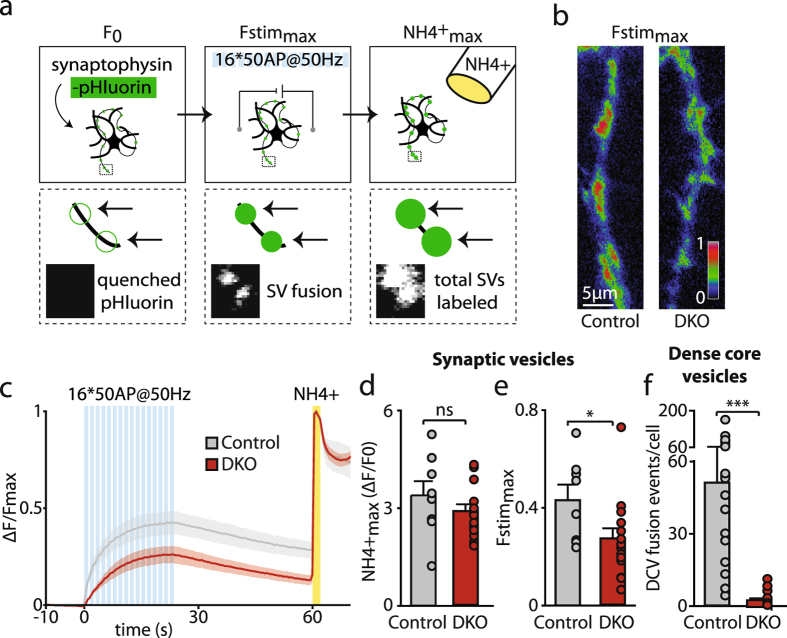
Intense stimulation in CAPS DKO neurons triggers relatively more SV than DCV exocytosis. (**a**) Schematic representation of the method to measure SV exocytosis with synaptophysin-pHluorin (SypHy). Neurons infected with SypHy (left) were stimulated (16 trains of 50 AP at 50 Hz; blue bars) to elicit SV exocytosis, which was detected by appearance of fluorescent puncta (middle). NH_4_^+^ supersfusion revealed the total pool of SypHy labeled SVs (right). Lower panels show a zoom of the dotted box in the upper panel. (**b**) Representative maximal SypHy response during stimulation (Fstim_max_) in a neurite of CAPS-2 KO (control) and CAPS DKO neurons. (**c**) ΔF/Fmax SypHy signal before, during and after stimulation (blue bars) and during and after NH_4_^+^ superfusion (yellow bar) in CAPS DKO and control neurons. Shaded area represents SEM. (**d**) NH_4_^+^_max_ (maximal ΔF/F0 during NH_4_^+^ wash) in control and CAPS DKO neurons. Mann-Whitney U test: p = 0.24 (not significant, ns). (**e**) Fstim_max_ (peak in the ΔF/Fmax SypHy graph, see c) of control and CAPS neurons. Mann-Whitney U test: p = 0.042 (*). (**f**) Average DCV exocytosis events in control and CAPS DKO neurons. Data from Fig. 2i, duplicated for clarity. Mann-Whitney U test: p = 2.0 *10^−8^ (***). Detailed information (average, SEM, n and detailed statistics) is shown in Table S1.

To assess the relative importance of CAPS-1 in DCV versus SV exocytosis, which is subject to a long standing debate^2, 4, 14, 30, 31^, we studied SV exocytosis in CAPS DKO neurons under identical conditions as for DCV exocytosis (Fig. 2). We infected neurons with synaptophysin-pHluorin (SypHy), which is targeted to SVs and the intravesicular pHluorin (pH sensitive GFP) is quenched at luminal pH (±pH 5.5)^32^. Electrical stimulation induced SV exocytosis and dequenched SypHy, detected by a gradual fluorescence increase. Brief superfusion with NH_4_^+^ instantly dequenched all SypHy and was used to visualize the total pool of SypHy labeled SVs in the synaptic terminals (Fig. 3a). Both CAPS DKO and control neurons showed SV exocytosis upon electrical stimulation (Fig. 3b,c). The total pool of SypHy labeled SVs, measured by the maximal NH_4_^+^ response, was unaltered between the two groups (control: 3.4 ± 0.45, CAPS DKO: 2.9 ± 0.21, all data and statistics in Table S1, Fig. 3d). The maximal response during stimulation (Fstim_max_), a measure for the total number of SVs that fused, was significantly lower in CAPS DKO neurons as compared to controls (control: 0.43 ± 0.06, CAPS DKO: 0.27 ± 0.04, all data and statistics in Table S1, Fig. 3c,e). The effect size for SV exocytosis, a 39% lower response, was smaller than for DCV exocytosis, a 96% lower response (t-test on the test statistics of DCV and SV exocytosis (for details see Materials and Methods): p = 4.9 *10^−20^ (***), all data and statistics in Table S1, Figs 2i and 3e,f). These data show that deletion of CAPS-1 expression has a larger effect on DCV exocytosis compared to SV exocytosis upon our intense electrical stimulation.

## Discussion

In this study we investigated which CAPS-1 domains are important for the function of CAPS-1 in mammalian dense core vesicle exocytosis. Synaptic localization was decreased after truncation of the C-terminus, but not by mutating the C2 or PH domain. Expression of none of the CAPS-1 mutants supported efficient DCV exocytosis in CAPS DKO neurons. In addition, during intense stimulation, DCV exocytosis was decreased by 96% while SV exocytosis decreased by 39% in CAPS DKO neurons.

We show that two point mutations in the C2 domain (K428E and G476E) do not alter the synaptic localization of CAPS-1, but do abolish most DCV exocytosis. CAPS-1 has two calcium binding affinities (K_D_ = 270 µM, K_D_ = 4.3 µM)^5^, but the C2 domain does not contain the conserved aspartic acid residues that coordinate C2 domain calcium binding^33^ and might therefore not bind calcium. Instead, the C2 domain is reported to regulate dimerization and C2 domain mutations alter the level of homo-dimerization: K428D (similar to K428E used here) decreases and G476E increases the percentage of CAPS-1 dimer^17^. Munc13 proteins, which share several domains with CAPS proteins (in addition to the C2 domain, also the MUN domain^20^) and also regulate DCV and SV exocytosis^29, 34^, also form homo-dimers via their C2A domain^35^. Homo-dimerization of Munc13 proteins is disrupted by K32E substitution^35, 36^, a mutation which corresponds to K428E in CAPS-1^17^. Similar to the situation for CAPS-1, alterations in the level of Munc13-2 dimerization also do not affect synaptic localization^36^. Disruption of the Munc13 protein dimer, mediated by the active zone scaffolding protein RIM, is required for Munc13 function in exocytosis^35–37^. Alternatively, mutating the C2 domain of CAPS or Munc13 proteins might impair a functional interaction between these proteins^12, 14^, resulting in the observed defects in exocytosis. In conclusion, Munc13 homo-dimerization inhibits its function, while mutations in CAPS-1 that are reported to increase or decrease dimerization both inhibit CAPS-1 function.

The CAPS-1 mutant with a C-terminal truncation did not support neuronal DCV exocytosis, in line with data from *C. elegans* and PC12 cells^21, 25^, and lacked synaptic enrichment. The availability of CAPS-1 at synapses increases DCV release probability^8^ and the lack of synaptic enrichment after C-terminal truncation may therefore explain the poor support of DCV exocytosis for this mutant. The lack of synaptic enrichment was only detected with the C-terminal truncation mutant. This suggests that the C-terminal domains of CAPS-1 are involved in the most upstream step of DCV exocytosis and that the C2 and PH domain, which are also essential for DCV exocytosis, are involved in more downstream steps. Surprisingly, the naturally occurring CAPS-2 isoform CAPS-2e, which aligns completely with the CAPS-1 C-terminal truncation mutant used here (C2 and PH domain are present but not the C-terminal domains), rescued DCV release in CAPS DKO chromaffin cells and EPSC amplitude in CAPS DKO neurons^10^. CAPS-1 and CAPS-2 both regulate exocytosis^8, 9, 12, 14^ and are 75% identical at the amino acid level. CAPS-1 is larger, including additional amino acids in the MHD1 and DCV domain. While six CAPS-2 splice isoforms have been reported^38^, no similar splicing isoforms have been described for CAPS-1. In addition, the CAPS-2e isoform ends with an additional, unique exon^38^, which is not present in CAPS-1. CAPS-1 is expressed equally in most brain regions but CAPS-2 is expressed in specific cell populations, most prominently in the cerebellum^6, 39^. During development, CAPS-2 expression is stable but CAPS-1 expression increases until postnatal day 21^6, 11^. Hence, despite high similarities, there are clear indications for functional differences between CAPS-1 and CAPS-2. Also, exceptional high expression by Semliki forest virus may have contributed to the rescue capacity of CAPS-2e^10^.

Since the C-terminal truncation removes the MHD1 and DCV domain, synaptic enrichment of CAPS-1^8^ is probably regulated by these domains. The MHD1 domain regulates binding of CAPS-1 to SNARE proteins^21^, which are also enriched at the synapse^40^. The DCV domain regulates CAPS-1 interaction with DCVs. Removal of 135 amino acids of this domain was shown to impair CAPS-1 interaction with DCVs and produce diffuse CAPS-1 expression in PC12 cells^16, 23^. However, in hippocampal neurons DCVs are not enriched at presynaptic terminals relative to other parts of the axon (although exocytosis occurs predominantly at the synapses)^29^. Furthermore, in a previous study on primary mouse neurons, the same neurons as used in the current study, we found no evidence for co-transport of CAPS-1 on DCVs^8^. Therefore, the interaction of the CAPS-1 MHD1 domain with SNARE proteins appears to be the most likely explanation for the C-terminal dependent synaptic enrichment of CAPS-1. In conclusion, CAPS-1 enrichment at the synapse is regulated by its C-terminus where it supports DCV exocytosis, which is different compared to CAPS-2, which supports DCV exocytosis in chromaffin cells without its C-terminus.

After CAPS’ initial discovery^41^, the protein was found to regulate DCV exocytosis in PC12 cells and melanotrophs^5, 7, 42^, DCV, but not SV, exocytosis in synaptosomes^31^ and to localize to DCVs, but not SVs, in brain homogenate^30^. Therefore, CAPS was initially considered to be specifically involved in DCV exocytosis and “not required for exocytosis of glutamate-containing vesicles”^31^. Currently, CAPS is still considered to “specifically regulate DCV release”^43^. Studies in *C. elegans* using intense stimulation (high K^+^ for 30–60 minutes) confirmed CAPS’ role in DCV exocytosis, while SV exocytosis was unaffected^2^. On the other hand, *Drosophila* dCAPS KO show defects in SV exocytosis upon mild stimulation (single action potential)^4^ and CAPS DKO mouse neurons show severe defects in both DCV (upon intense stimulation)^8^ and SV (upon mild stimulation)^14^ exocytosis. The SV exocytosis defect in CAPS DKO neurons were partly overcome after intense stimulation or high intracellular calcium. Therefore, the authors of the latter study argued that the absence of SV exocytosis phenotype in *C. elegans* could be explained by massive calcium influx upon intense stimulation^14^. Alternatively, it has been proposed that the decreased SV exocytosis might be secondary to the chronic inhibition of DCV exocytosis and the consequent reduction in ambient neuropeptides/neuromodulators in (developing) CAPS DKO neurons^4^. However, acute CAPS-1 expression in CAPS DKO neurons fully restored (rescued) SV exocytosis^14^, neurons were cultured in neuropeptide rich medium and no developmental defects were detected (see ref. 14 for further discussion on this topic). To add to this unresolved issue, we studied DCV and SV exocytosis in CAPS DKO neurons under identical conditions of intense stimulation (16 trains of 50 action potentials at 50 Hz). Using this paradigm, DCV exocytosis is much more affected (96% inhibition) compared to SV exocytosis (39% inhibition). Our data confirms that during conditions of high calcium influx, CAPS’ function in SV, but not DCV exocytosis, becomes partly redundant.

CAPS and Munc13 proteins both regulate exocytosis, probably at a step upstream of the actual fusion (priming)^8, 14, 29, 34^. Overexpression of Munc13–1 in chromaffin cells increases DCV exocytosis with 300%^44^. However, overexpression of Munc13-1 on a CAPS DKO background does not increase exocytosis in chromaffin cells or rescue the loss of SV or DCV exocytosis^12, 14^. This indicates that CAPS is required for the stimulatory effect of Munc13-1 and the two proteins have non-redundant functions. Deletion of *unc-13*/*munc13-1/2* expression abolishes SV exocytosis in *C. elegans* and mammalian neurons^2, 3, 45^. In *C. elegans*, intense stimulation, when CAPS’ function becomes partly redundant, does not rescue SV exocytosis in UNC-13 mutants^2^. Conversely, DCV exocytosis is 60% reduced in mammalian Munc13-1/2 DKO neurons^29^ and is not affected in *C. elegans* ^2, 3^. Hence, while CAPS are partially redundant for SV exocytosis but almost essential for DCV exocytosis, the opposite is true for Munc13: (partially) redundant for DCV fusion, but essential for SV exocytosis. Possibly, the PH domain, which is present only in CAPS^20^ and essential for DCV exocytosis, contributes to the difference between CAPS and Munc13 for DCV exocytosis.

## Methods

### Plasmids

Mouse CAPS-1 (CAPS-1-ires-EGFP) was previously described^8^. For CAPS-1 (K428E), CAPS-1 (G476E)^22^, CAPS-1 (R558D/K560E/K561E)^16^ and CAPS-1 (Δ654–1355), mutations were generated in CAPS-1 (mKIAA1121-Kazusa DNA) and sequence verified to obtain mutant CAPS-1 construct with an IRES-EGFP. NPY-mCherry was generated by replacing Venus in NPY-Venus^46^ with mCherry. Synaptophysin-pHluorin (SypHy) was described before^32^.

### Laboratory animals, primary neuron cultures and infection

All animal experiments were approved by the animal ethical committee of the VU University/VU University Medical Centre (“Dier ethische commissie (DEC)”; license number: FGA 11-03). Animals were housed and bred according to institutional and Dutch governmental guidelines and regulations.

CAPS DKO embryonic day 18 (E18) embryos were acquired by caesarean section of pregnant mice^14^. Primary neuron cultures from CAPS DKO and CAPS-2 KO control littermates were prepared as described before^8, 47^. Briefly, dissected hippocampi and cortices were digested with 0.25% trypsin (Life Technologies) in Hanks’ balanced salt solution (Sigma) with 10 mM HEPES (Life Technologies) for 20 min at 37 °C. Hippocampi were washed, triturated and 1,000–2,000 neurons/well were plated on pre-grown micro-islands generated by plating 6000 rat glia on 18mm glass coverslips coated with agarose and stamped with a solution of 0.1 mg/ml poly-D-lysine (Sigma) and 0.7 mg/ml rat tail collagen (BD Biosciences) as in Mennerick *et al*. (1995) and Wierda *et al*.^48, 49^. For western blots, cortices were washed, triturated and 300,000 neurons/well were plated on 6 well plates coated with a solution of 0.5*10^−3^% poly-L-ornithin and 2.5 µg/ml laminin (Sigma). Neurons were kept in neurobasal medium supplemented with 2% B-27, 18 mM HEPES, 0.25% glutamax and 0.1% Pen-Strep (Life Technologies) at 37 °C and 5% CO_2_. Infection of neurons with lentiviral particles was at day *in vitro* (DIV) 0–2 (CAPS-1), DIV 4–10 (NPY-mCherry) and DIV 8 (SypHy).

### Imaging

Neurons were imaged at DIV 9–14 in Tyrode’s solution (2 mM CaCl_2_, 2.5 mM KCl, 119 mM NaCl, 2 mM MgCl_2_, 30 mM glucose, 25 mM HEPES; pH 7.4). Imaging was performed with a custom build microscope containing an imaging microscope (AxioObserver.Z1), 561 nm and 488 nm lasers, appropriate filter sets, 40x oil objective (NA 1.3) and an EM-CCD camera (C9100-02; Hamamatsu, pixel size 200 nm). Images were acquired with AxioVision 4.8 software (Zeiss) at 2 Hz. Electrical stimulation was performed with two parallel platinum electrodes placed around the neuron. 16 trains of 50 action potentials at 50 Hz were initiated by a Master-8 (AMPI) and a stimulus generator (A-385, World Precision Instruments) delivered the 1ms pulses of 30 mA. Tyrode’s with 50 mM NHCl_2_ (replacing 50 mM NaCl), used in the SypHy experiments, was delivered by gravity flow through a capillary placed above the neuron. Experiments were performed at room temperature (21–25 °C).

### Fixation and immunocytochemistry

Neurons were fixed at DIV 10–14 (for CAPS-1 levels and localization) or at DIV 2, 4, 7, 14 (for neuronal development) in 2% formaldehyde (Merck) in phosphate-buffered saline (PBS; 137 mM NaCl, 2.7 mM KCl, 10 mM Na_2_HPO_4_, 1.8 mM KH_2_PO_4_, pH 7.4) for 10 minutes followed by 4% formaldehyde in PBS for 30 minutes. Cells were permeabilized in 0.5% TritonX-100 (Fisher Chemical) for 5 minutes and blocked with 0.1% TritonX-100 and 2% normal goat serum for 30 minutes. Primary antibody incubation polyclonal CAPS-1 (SySy; 1:200), polyclonal MAP2 (Abcam; 1:1000), polyclonal synaptophysin 1 (SySy; 1:1000), monoclonal SMI312 (Biolegend; 1:1000), polyclonal synaptotagmin 1 (W855 a kind gift from T. Südhof, Stanford, CA; 1:2000) was performed over night at 4 °C. Alexa Fluor conjugated secondary antibodies (1:1000; Invitrogen) were incubated for 1 hour at room temperature. Coverslips were mounted in Mowiol and imaged on a Zeiss LSM 510 confocal laser-scanning microscope (40x objective; NA 1.3) and LSM510 software.

### Western blotting

Cortical neurons were lysed at DIV 10–11. Lysates were run on a SDS-PAGE and transferred to a Polyvinylideenfluoride (PVDF) membranes (Bio-rad). Membranes were blocked with 5% milk (Merck) in PBS with 0.1% Tween-20, and subsequently incubated with polyclonal CAPS-1 (SySy; 1:500) and monoclonal actin (Chemicon; 1:10.000) antibodies over night (4 °C). Secondary alkaline phosphatase conjugated antibodies (1:10.000, Jackson Immuno Research) were incubated for 30 minutes (4 °C), visualized with AttoPhos (Promega) and scanned with a FLA-5000 fluorescent image analyzer (Fujifilm).

### Data analysis

To determine enrichment of CAPS-1 (or VAMP2 as a control) in overlap with synaptophysin 1 puncta, fluorescence images were analyzed using the ImageJ plugin JACoP^50^. Colocalization of VAMP2 in synaptophysin 1 was used as a reference to be able to quantitatively compare data of multiple independent experiments and perform statistics. Colocalization of CAPS-1 in synaptophysin 1 was calculated relative to this positive control (“relative Manders’ coefficient”). Neurite length, synaptotagmin 1 puncta number, number and Sholl analysis of DCVs were analyzed with SynD^51^ software running in MATLAB (MathWorks, Inc.). CAPS-1 positive puncta were counted manually in ImageJ. Westernblots were analyzed using Image Studio Lite (LI-COR). Analysis of DCV exocytosis events was performed manually: abrupt disappearance of NPY-mCherry positive puncta was detected with ImageJ, which was used to calculate number and timing of exocytosis events. Release probability was calculated by dividing the number of exocytosis events by the total number of DCVs. SV exocytosis was measured with ImageJ in manually placed regions where NH_4_^+^ increased fluorescence.

### Statistics

To test multiple groups (Figs 1 and 2), we used one-way analysis of variance (ANOVA) followed by a post-hoc Dunnett’s test to compare conditions to the control condition (CAPS2 KO control neurons if not specified otherwise) if the ANOVA showed significant differences. To test two groups (Fig. 3) we used Mann-Whitney U test, because of small sample size and outliers in the dataset. To compare the data from Fig. 3e and f, we used a t-test to assess whether the U-values gained with the Mann-Whitney U tests are significantly different (standard errors were pooled). We used a two-way ANOVA to test multiple groups with two variables (DIV and genotype, Figure S1), followed by a post-hoc Tukey test. Data is represented as average with standard error of the mean (SEM). Dots in bar graphs indicate individual data points of single neurons.

### Data availability

Data generated or analyzed during this study are included in this published article (and its Supplementary Information files). Detailed datasets are available from the corresponding author on request.

## Electronic supplementary material

Supplementary information
