# Supplementary material for: CAPS-1 requires its C2, PH, MHD1 and DCV domains for dense core vesicle exocytosis in mammalian CNS neurons
Source: Sci Rep. 2017 Sep 7;7:10817. doi: 10.1038/s41598-017-10936-4 (PMC5589909; doi:10.1038/s41598-017-10936-4)
Supplement: Supplementary file 1 — Supplementary information [file 41598_2017_10936_MOESM1_ESM.pdf]

Supplementary files

CAPS-1 requires its C2, PH, MHD1 and DCV domains for dense core vesicle exocytosis in mammalian CNS neurons

Linda van Keimpema[1,2], Robbelien Kooistra[1], Ruud F. Toonen[1 #], and Matthijs Verhage[1 #]

[1] Department of Functional Genomics, Center for Neurogenomics and Cognitive Research, VU University, 1081 HV Amsterdam, The Netherlands

[2] Sylics (Synaptologics BV), PO box 71033, 1008 BA Amsterdam, The Netherlands

[#] To whom correspondence should be addressed: Matthijs Verhage or Ruud F. Toonen, Center for Neurogenomics and Cognitive Research, VU University, De Boelelaan 1087, 1081 HV Amsterdam, The Netherlands. Email: m.verhage@vu.nl or r.f.g.toonen@vu.nl

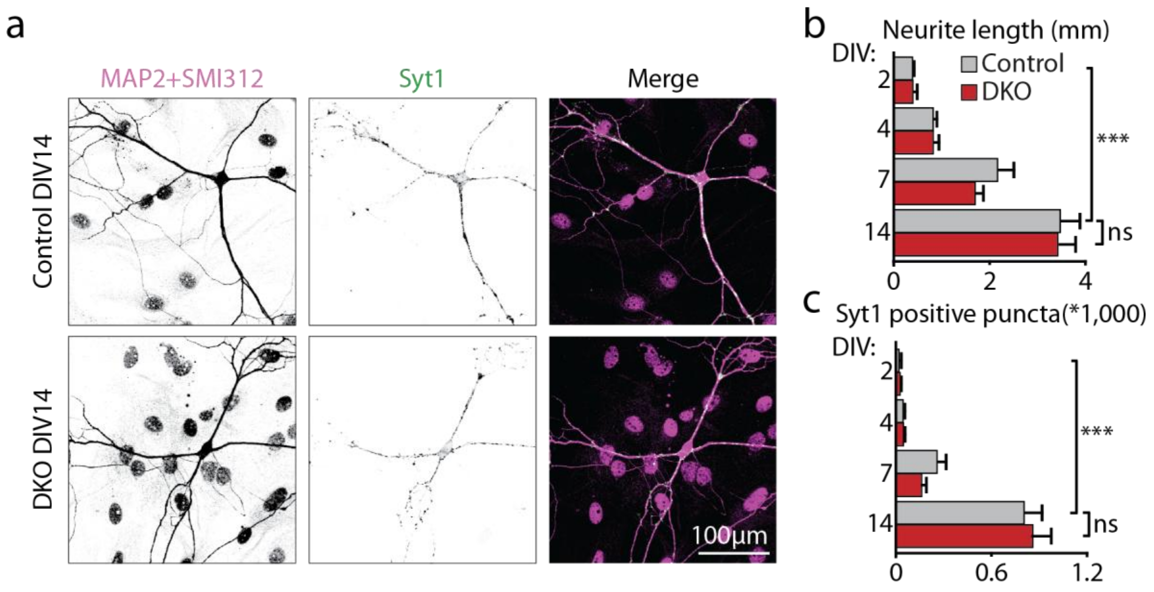

Figure S 1 – CAPS DKO neurons show no developmental defects

a: Representative images of CAPS-2 KO (control, top) and CAPS DKO (bottom) neurons at DIV 14, stained with dendrite marker (MAP2; magenta) and axonal marker (SMI312; magenta) and synaptotagmin 1 (syt1, green).

b: Total neurite length in mm of control and CAPS DKO neurons at DIV 2, 4, 7 and 14 in CAPS DKO and control neurons. 2-way ANOVA: genotype: p = 0.53 (not significant, ns), DIV: p = 7.8 $*10^{-25}$ (***), genotype*DIV: p = 0.72 (ns).

c: Number of syt1 positive puncta in control and CAPS DKO neurons at DIV 2, 4, 7 and 14. 2-way ANOVA: genotype: p = 0.85 (ns), DIV: p = 9.0 $*10^{-28}$ (***), genotype*DIV: p = 0.71 (ns).

Detailed information (average, SEM, n and detailed statistics) is shown in Table S1.

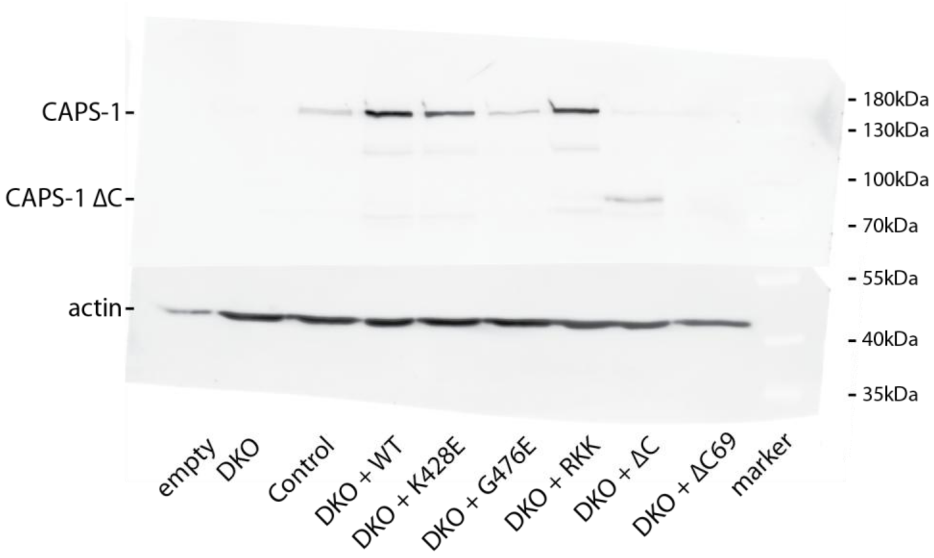

Figure S 2 – Western blot of CAPS DKO cortical neurons

Uncropped western blot of CAPS DKO cortical neurons infected with wild type or mutant CAPS-1 constructs and CAPS-2 KO control neurons (Figure 1b shows cropped blot). DKO + Δ69 are CAPS DKO cortical neurons infected with a mutant lacking the last 69 amino acids of the C-terminus [1], which was not further used in this study because of strongly reduced expression levels.

Supplementary References

1. Parsaud, L. et al. Calcium-dependent activator protein for secretion 1 (CAPS1) binds to syntaxin-1 in a distinct mode from Munc13-1. J Biol Chem 288, 23050–23063, doi:10.1074/jbc.M113.494088 (2013).

Table S 1 – Data and statistics

For each data figure, the measured variable, tested groups, average and SEM, n (independent cells) and N (independent weeks) are listed. Statistical tests were performed on all groups, unless specified otherwise. Statistical tests used $\alpha = 0.05$ and were two-tailed. *: $p<0.05$; **: $p<0.01$; ***: $p<0.001$. N.A.: not applicable.

Figure	measured variable	group	average ± SEM	n (N)	statistics
1c	relative CAPS-1 level	CAPS DKO	0.05 ± 0.04	2	N.A.
		control	1.00 ± 0.74	2	
		DKO + WT	5.25 ± 2.32	2	
		DKO + K428E	3.62 ± 0.47	2	
		DKO + G476E	2.08 ± 0.79	2	
		DKO + RKK	7.60 ± 0.55	2	
		DKO + ΔC	2.91 ± 0.15	2	
1e	relative Manders' coefficient	control	0.48 ± 0.05	24 (4)	1-way ANOVA (CAPS conditions):
		DKO + WT	0.61 ± 0.05	25 (4)	p = 0.067 (ns)
		DKO + K428E	0.66 ± 0.07	9 (2)	
		DKO + G476E	0.48 ± 0.12	10 (2)	
		DKO + RKK	0.64 ± 0.08	14 (2)	
		DKO + ΔC	0.31 ± 0.05	5 (2)	
		VAMP	1.00 ± 0.03	26 (4)	
1f	Number CAPS-1 puncta	control	34.8 ± 7.7	5 (2)	N.A.
		DKO + ΔC	0.0 ± 0.0	5 (2)	
2b	number DCVs	control	1.9 ± 0.1 *10^3	18 (4)	1-way ANOVA:
		DKO	2.3 ± 0.2 *10^3	17 (6)	p = 0.42 (ns)
		DKO + WT	2.4 ± 0.2 *10^3	38 (6)	
		DKO + K428E	2.5 ± 0.3 *10^3	11 (3)	
		DKO + G476E	2.3 ± 0.3 *10^3	16 (3)	
		DKO + RKK	2.3 ± 0.3 *10^3	16 (3)	
		DKO + ΔC	2.8 ± 0.5 *10^3	13 (3)	
2i	DCV exocytosis events	control	51 ± 9.7	18 (4)	1-way ANOVA:
		DKO	2.1 ± 0.8	17 (6)	p = 5.9 *10^{-9} (***)
		DKO + WT	36.9 ± 6.3	38 (6)	Post-hoc Dunnett's test (compared to control):
		DKO + K428E	4.3 ± 1.7	11 (3)	DKO: p = 2.0 *10^{-6} (***); DKO + WT: p = 0.27 (ns)
		DKO + G476E	6.4 ± 1.9	16 (3)	DKO + K428E: p = 8.5 *10^{-5} (***); DKO + G476E: p = 2.7 *10^{-5} (***)
		DKO + RKK	8.8 ± 3.8	16 (3)	DKO + RKK: p = 7.4 *10^{-5} (***); DKO + ΔC: p = 5.2 *10^{-5} (***)
		DKO + ΔC	5.4 ± 2.7	13 (3)	Post-hoc Dunnett's test (compared to DKO):
					DKO + K428E: p = 1.0 (ns); DKO + G476E: p = 1.0 (ns)
					DKO + RKK: p = 0.96 (ns); DKO + ΔC: p = 1.0 (ns)
2j	DCV release probability	control	2.9 ± 0.55 %	18 (4)	1-way ANOVA:
		DKO	0.11 ± 0.05 %	17 (6)	p = 2.2 *10^{-8} (***)
		DKO + WT	1.8 ± 0.33 %	38 (6)	Post-hoc Dunnett's test (compared to control):
		DKO + K428E	0.20 ± 0.07 %	11 (3)	DKO: p = 1.0 *10^{-6} (***); DKO + WT: p = 0.045 (*)
		DKO + G476E	0.31 ± 0.10 %	16 (3)	DKO + K428E: p = 3.2 *10^{-5} (***); DKO + G476E: p = 8.0 *10^{-6} (***)
		DKO + RKK	0.43 ± 0.18 %	16 (3)	DKO + RKK: p = 2.5 *10^{-5} (***); DKO + ΔC: p = 1.9 *10^{-4} (***)
		DKO + ΔC	0.41 ± 0.22 %	13 (3)	Post-hoc Dunnett's test (compared to DKO):
					DKO + K428E: p = 1.0 (ns); DKO + G476E: p = 1.0 (ns)
					DKO + RKK: p =0.98 (ns); DKO + ΔC: p = 0.98 (ns)

Figure	measured variable	group		average ± SEM	n (N)	statistics	
3d	NH4+$_{max}$	control		3.4 ± 0.45	8 (2)	Mann-Whitney U test	
		CAPS DKO		2.9 ± 0.21	14 (2)	p = 0.24 (ns)	
3e	Fstim$_{max}$	control		0.43 ± 0.06	8 (2)	Mann-Whitney U test	t-test
		CAPS DKO		0.27 ± 0.04	14 (2)	p = 0.042 (*)	(comparing U-values of
3f	DCV exocytosis events	control		51 ± 9.7	18 (4)	Mann-Whitney U test	3e (U = 26) and 3f (U = 7))
		CAPS DKO		2.1 ± 0.8	17 (6)	p = 2.0 *10^{-8} (***)	p = 4.9 *10^{-20} (***)
S1b	neurite length (mm)	Control	DIV 2	0.38 ± 0.03	20 (2)	2-way ANOVA:	
		CAPS DKO	DIV 2	0.41 ± 0.06	13 (2)	genotype: p = 0.53 (ns)	
		Control	DIV 4	0.81 ± 0.08	20 (2)	DIV: p = 7.8 *10^{-25} (***)	
		CAPS DKO	DIV 4	0.84 ± 0.09	20 (2)	genotype*DIV: p = 0.72 (ns)	
		Control	DIV 7	2.14 ± 0.33	17 (2)	Post-hoc Tukey test for DIV:	
		CAPS DKO	DIV 7	1.68 ± 0.17	19 (2)	DIV 2 vs 4: p = 0.33 (ns); DIV 2 vs 7: p = 2.2 * 10^{-7} (***)	
		Control	DIV 14	3.44 ± 0.41	20 (2)	DIV 2 vs 14: p = 4.4 *10^{-13} (***) ; DIV 4 vs 7: p = 1.4 *10^{-4} (***)	
		CAPS DKO	DIV 14	3.38 ± 0.37	20 (2)	DIV 4 vs 14: p = 4.4 *10^{-13} (***) ; DIV 7 vs 14: p = 4.2 *10^{-8} (***)	
S1c	syt1 positive puncta	Control	DIV 2	22 ± 4.6	20 (2)	2-way ANOVA:	
		CAPS DKO	DIV 2	27 ± 6.5	13 (2)	genotype: p = 0.85 (ns)	
		Control	DIV 4	45 ± 10.5	20 (2)	DIV: p = 9.0 *10^{-28} (***)	
		CAPS DKO	DIV 4	48 ± 9.4	20 (2)	genotype*DIV: p = 0.71 (ns)	
		Control	DIV 7	259 ± 57	17 (2)	Post-hoc Tukey test for DIV:	
		CAPS DKO	DIV 7	162 ± 31	19 (2)	DIV 2 vs 4: p = 0.99 (ns); DIV 2 vs 7: p = 0.04 (*)	
		Control	DIV 14	809 ± 112	20 (2)	DIV 2 vs 14: p= 4.4 *10^{-13} (***) ; DIV 4 vs 7: p= 0.07 (ns)	
		CAPS DKO	DIV 14	862 ± 117	20 (2)	DIV 4 vs 14: p= 4.4 *10^{-13} (***) ; DIV 7 vs 14: p= 4.4 *10^{-13} (**)	
